# Anatomical liver resection improves surgical outcomes for combined hepatocellular-cholangiocarcinoma: A propensity score matched study

**DOI:** 10.3389/fonc.2022.980736

**Published:** 2022-08-18

**Authors:** Wen-qiang Wang, Jian Li, Bin-yong Liang, Xing Lv, Rong-hua Zhu, Jin-lin Wang, Zhi-yong Huang, Shu-hong Yang, Er-lei Zhang

**Affiliations:** ^1^ Hepatic Surgery Center, Tongji Hospital, Tongji Medical College, Huazhong University of Science and Technology, Wuhan, China; ^2^ Department of Obstetrics and Gynecology, Tongji Hospital, Tongji Medical College, Huazhong University of Science and Technology, Wuhan, China

**Keywords:** anatomical resection, non-anatomical resection, combined hepatocellular carcinoma and cholangiocarcinoma, surgery, prognosis

## Abstract

**Background:**

The efficacies of anatomical resection (AR) and non-anatomical resection (NAR) in the treatment of combined hepatocellular-cholangiocarcinoma (cHCC-CCA) remain unclear. This study aimed to compare the prognostic outcomes of AR with those of NAR for cHCC-CCA.

**Method:**

Patients diagnosed with pathology-confirmed cHCC-CCA, and who underwent curative resection at Tongji hospital between January 2010 and December 2019 were included in this retrospective study. A one-to-one propensity score matching (PSM) analysis was used to compare the long-term outcomes of AR to those of NAR.

**Results:**

A total of 105 patients were analyzed, of whom 48 (45.7%) and 57 (54.3%) underwent AR and NAR, respectively. There were no significant differences in short-term outcomes between the two groups, including duration of postoperative hospital stay, the incidence of perioperative complications, and incidence of 30-day mortality. However, both, the 5-year overall survival (OS) and recurrence-free survival (RFS) rates of AR were significantly better than those of NAR (40.5% vs. 22.4%, *P*=0.002; and 37.3% vs. 14.4%, *P*=0.002, respectively). Multivariate analysis showed that NAR, multiple tumors, larger-sized tumors (>5 cm), cirrhosis, lymph node metastasis, and vascular invasion were independent risk factors for poor prognoses. Stratified analysis demonstrated similar outcomes following AR versus NAR for patients with tumors > 5cm in diameter, while AR had better survival than NAR in patients with tumors ≤5 cm in diameter. After PSM, when 34 patients from each group were matched, the 5-year OS and RFS rates of AR were still better than those of NAR.

**Conclusion:**

Patients with cHCC-CCA who underwent AR had better long-term surgical outcomes than those who underwent NAR, especially for those with tumors ≤5 cm in diameter. However, no differences in the risk of surgical complications were detected between the two groups.

## Introduction

Combined hepatocellular-cholangiocarcinoma (cHCC-CCA) is a rare type of primary liver cancer that exhibits both hepatocytic and cholangiocytic differentiation within the same tumor; cHCC-CCA has an incidence rate that ranges from 0.4–14.2% and is reported to be more common in men and those with chronic liver disease (1–3). cHCC-CCA is an aggressive malignancy, with clinical and biological patterns overlapping with those of hepatocellular carcinoma (HCC) and intrahepatic cholangiocarcinoma (iCCA) (1). Due to the low incidence of cHCC-CCA, there are few published studies (mostly with low sample sizes) on the treatment and prognosis of the condition (2, 4, 5). Furthermore, there are no detailed accounts of the clinical behavior, surgical outcomes, and prognostic factors for cHCC-CCA (5–7). Compared with HCC and iCCA, standardizing treatment for cHCC-CCA is difficult due to several factors. First, it is difficult to differentiate cHCC-CCA from HCC or iCCA through imaging. Second, the incidence of cHCC-CCA is relatively low, making it difficult for a single institution to have enough patients for detailed studies. The only curative option for patients with cHCC-CCA was found to be R0 resection with lymph node dissection; however, even after radical hepatectomy or liver transplantation, long-term survival remained low (2, 4, 8, 9). The 5-year tumor recurrence rate in cHCC-CCA patients was reported to be as high as 80%, and the 5-year overall survival (OS) rates were less than 30% (10–14). High incidence rates of postoperative recurrence in cHCC-CCA patients even after curative treatment is also a major issue in the treatment of this condition.

A nationwide study in China has indicated that although cHCC-CCA reflects the malignant behavior of iCCA, it should be characterized as a subtype of HCC due to similarities in mortality rates and long-term surgical outcomes between HCC and cHCC-CCA (15). The superiority of anatomical resection (AR) over non-anatomical resection (NAR) for surgical outcomes in HCC patients is an ongoing controversy. Since cHCC-CCA has characteristics of both HCC and iCCA, the tumors have a high propensity to invade intrahepatic pedicle structures, which allows the tumor to spread *via* the closest portal veins or bile ducts. Therefore, the complete removal of tumor-bearing hepatic pedicles is considered to be ideal for surgical eradication of potential micrometastases (16). Theoretically, AR in patients with cHCC-CCA could reduce the risk of local recurrence and may improve patient survival (17). However, no reports have proved that AR is superior to NAR in treating cHCC-CCA as yet.

Therefore, this study was undertaken to clarify which—AR or NAR—is the superior treatment option based on short-term and long-term outcomes for patients with cHCC-CCA.

## Patients and methods

### Study population and data collection

**Figure 1:**
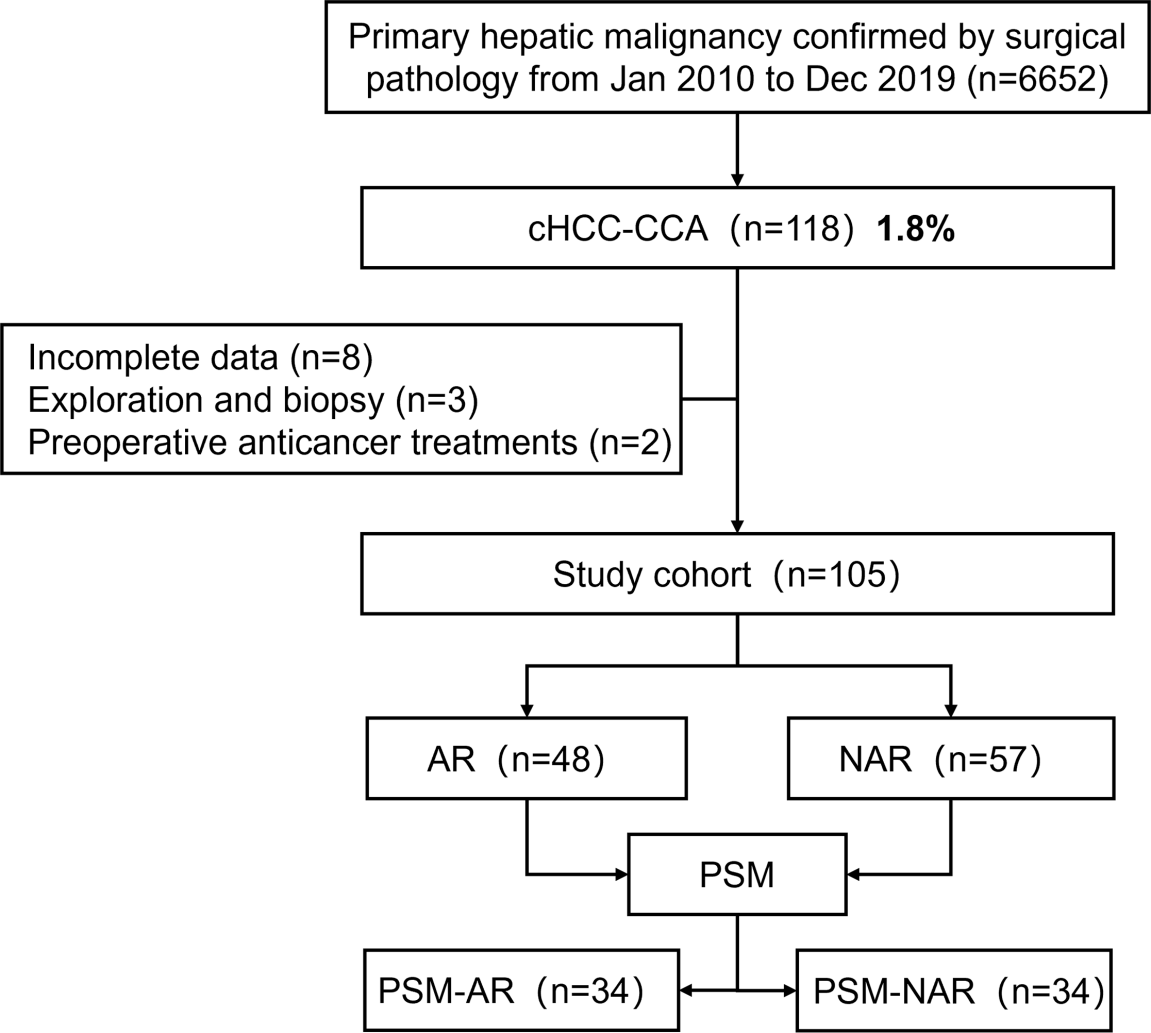
Flowchart of steps taken for patient selection for this study. A*R*, anatomical resection; *NAR*, non-anatomical resection; *PSM*, propensity score matching.

Of the 6652 patients who underwent hepatectomy for primary hepatic malignancy between January 2010 and December 2019 at the Hepatic Surgery Center, Tongji Hospital, 118 (1.8%) were identified as having pathology-confirmed cHCC-CCA. Of these, eight were excluded due to incomplete data (including six patients who were lost to follow-up), three were excluded as exploration and biopsies confirmed that the tumors were not cHCC-CCA, and two were excluded as they had received preoperative anticancer treatments (**Figure 1**). The remaining 105 patients were divided into NAR (n=57) and AR groups (n=48) according to the hepatic resection they underwent.

Demographic and clinical data including age, sex, Eastern Cooperative Oncology Group-Performance status (ECOG-PS), Child classification, presence of underlying liver disease, positivity for hepatitis B viral surface antigen (HBsAg) and hepatitis C viral antibody (HCV-Ab), liver function, complete blood count, coagulation profile, tumor markers including serum α-fetoprotein (AFP), carcinoembryonic antigen (CEA) and carbohydrate antigen 19-9 (CA19-9) levels were collected. Histopathological factors including the tumor size and number, vascular invasion, lymph node metastasis (LNM), and tumor stage according to the 8^th^ edition of the Union for International Cancer Control TNM classification (8^th^ TNM stage) were also recorded (18).

### Surgical procedures

The main surgical procedure for AR involved complete identification of the target Couinaud segment(s), following which parenchymal dissection was performed along the segmental border. Next, landmark veins were exposed on the cut surface of the liver, and the corresponding portal branches were ligated for trisectionectomy, hemihepatectomy, sectionectomy, and segmentectomy (19). For NAR (also known as conventional limited resection), the surgical procedure focused on tumor resection with a negative tumor margin regardless of segment or section anatomy. Postoperative morbidity was defined as the occurrence of complications during the hospital stay or within 3 months of resection. Complication severity was graded as per the Clavien–Dindo classification system (20).

### Follow-up

Postoperative follow-up consisted of abdominal ultrasound, computed tomography (CT), or magnetic resonance imaging (MRI) along with laboratory tests to check liver function. These included checking the levels of α-fetoprotein (AFP), carbohydrate antigen 19-9 (CA19-9), and carcinoembryonic antigen (CEA) every 2–3 months during the first 2 years after surgery, and then every 4–6 months thereafter. Follow-up data were collected until February 28, 2022. Recurrence-free survival (RFS) was defined as the period after the operation when no tumor recurrence could be detected by imaging or biopsy. Overall survival (OS) was the time interval between the surgery and date of death (if any).

### Propensity score matching analysis

Propensity score matching (PSM) was performed to reduce biases arising from the different distributions of covariates among patients who underwent AR and those who underwent NAR. Of all the variables identified, several were significantly and independently different between the two groups. Based on these results, the following variables were included in the 1:1 PSM analysis: CEA, prothrombin time (PT), white blood cell (WBC) count, and presence of solitary tumor. To achieve the highest homogeneity, the caliper was set to 0.10.

### Statistical analysis

For continuous variables, medians with inter-quartile ranges (IQR) have been reported. Such variables were compared using independent sample t-tests or Mann-Whitney U tests. Categorical variables were expressed as frequencies or percentages and compared using the Chi-square test or Fisher’s exact test. Kaplan-Meier (K-M) survival curves were used to compare survival rates between the AR and NAR groups using the log-rank test. Potential risk factors associated with OS and RFS were identified using univariate and multivariable Cox hazard regression models, and all variables with P<0.050 in the univariate analyses were utilized in multivariate analyses to determine independent risk factors. For all tests, P< 0.050 was considered statistically significant. All analyses were performed using the SSPS 24.0 software (IBM Corp., Armonk, NY, USA).

## Results

### Demographic and clinicopathologic characteristics

Of the 105 patients with cHCC-CCA included in this study, there were 90 (85.7%) men and 15 (14.3%) women; the mean age of the patients was 53 years (range, 28–83 years). Details regarding patient demographics, preoperative procedures, tumor characteristics, and operative procedures and care are reported in **Table 1**. A total of 57 patients underwent NAR (54.3%) while 48 underwent AR (45.7%). There were substantial differences in background variables between the two groups before PSM analysis. Patients in the AR group had significantly higher CEA levels and WBC counts, along with lower PT levels and smaller tumors than those in the NAR group. There were no significant differences in other clinicopathologic characteristics between the two groups. Details of the surgical procedures that the 48 patients who underwent AR are as follows: trisectionectomy (n=2); hemihepatectomy (n=11); sectionectomy (n=9); segmentectomy (n=16); combined resection of segments (n=10).

**Table 1:** Baseline patient characteristics.

Variable	Before PSM			After PSM		
	NAR (N=57)	AR (N=48)	*P* value	PSM-NAR (N=34)	PSM-AR (N=34)	*P* value
**Demographics**						
Age, median (IQR), y	52(48-60)	53(46-60)	0.393	52(46-60)	52(46-57)	0.870
Sex ratio, Male: Female	48:9	42:6	0.631	29:5	30:4	1.000
**Preoperative variables**						
ECOG-PS, 0:1	49:8	43:5	0.768	29:5	31:3	0.709
HBsAg-positive, n (%)	36(63.2)	30(62.5)	0.945	21(61.8)	23(67.6)	0.612
HCVAb-positive, n (%)	2(3.5)	0(0)	0.499	2(3.5)	0(0)	0.499
AFP, median (IQR), μg/L	97(24-391)	74(12-502)	0.379	95(24-231)	212(12-848)	0.230
CEA, median (IQR), μg/L	3.5(2.0-4.1)	4.3(3.3-7.2)	**0.021**	3.6(2.7-4.1)	3.7(2.9-4.8)	0.882
CA-199, median (IQR), U/L	27(8-60)	37(9-75)	0.746	27(8-77)	16(6-67)	0.440
ALT, median (IQR), U/L	25(18-39)	23(18-36)	0.844	23(17-36)	28(22-30)	0.764
TBIL, median (IQR), μmol/L	10.5(7.5-15.6)	11.0(8.6-14.0)	0.827	10.7(7.2-15.1)	10.2(7.6-13.2)	0.448
ALB, median (IQR), g/L	40.2(36.4-42.9)	39.9(36.4-43.6)	0.842	40.3(36.8-44.2)	40.2(36.5-44.3)	0.844
PT, median (IQR), s	13.6(12.9-14.3)	13.1(12.8-14.0)	**0.035**	13.5(12.8-14.3)	13.1(12.9-14.0)	0.273
WBC, median (IQR), *10^9/L	5.5(4.4-6.5)	6.2(4.8-7.3)	**0.027**	5.7(4.6-6.8)	6.0(4.5-6.9)	0.756
HB, median (IQR), g/L	129(118-148)	136(124-147)	0.515	130(122-145)	136(123-147)	0.655
PLT, median (IQR), *10^9/L	184(128-237)	188(138-231)	0.868	181(123-236)	196(141-227)	0.868
Child-Pugh Class, A: B	54:3	45:3	0.828	31:3	31:3	1.000
Splenomegaly, n (%)	16(28.1)	11(22.9)	0.547	10(29.4)	9(26.5)	0.787
**Tumor and operative variables**						
Size, median (IQR), cm	5.7(3.5-8.3)	5.0(3.5-7.8)	0.771	6.0(3.5-9.2)	5.6(3.4-8.0)	0.532
Solitary, n (%)	40(70.2)	42(87.5)	**0.033**	27(79.4)	28(82.4)	0.758
Laparoscopic surgery, n (%)	7(12.3)	10(20.8)	0.236	2(5.9)	9(26.5)	**0.045**
Operation time, median (IQR), min	180(160-200)	185(155-230)	0.105	180(160-200)	195(160-230)	0.058
Blood loss, median (IQR), ml	250(200-350)	300(200-475)	0.327	300(200-500)	300(200-450)	0.561
Blood transfusion, n (%)	6(10.5)	3(6.3)	0.504	4(11.8)	3(8.8)	1.000
Positive margin, n (%)	5(8.8)	2(2.1)	0.450	2(5.9)	2(5.9)	1.000
Differentiation, well/moderate: poor	45:12	39:9	0.811	28:6	27:7	0.758
Vascular invasion, n (%)	12(21.1)	7(14.6)	0.391	7(20.6)	7(20.6)	1.000
Lymph node metastasis, n (%)	5(8.8)	3(6.3)	0.724	4(11.8)	1(2.9)	0.356
8^th^AJCC TNM staging, I: II: III	32:20:5	34:11:3	0.298	33:19:5	34:11:3	0.389

PSM, propensity score matching; NAR, non-anatomical resection; AR, anatomical resection; IQR, interquartile range; HBsAg, hepatitis B surface antigen; HCV, hepatitis C virus; AFP, α-fetoprotein; CEA, carcinoembryonic antigen; CA19-9, carbohydrate antigen 19-9; ALT, alanine transaminase; TBIL, total bilirubin; ALB albumin, PT, prothrombin time; WBC, white blood cell; HB, hemoglobin; PLT, platelet. Bold values: statistically significant P values.

### Postoperative outcomes

The overall incidence rates of postoperative complications and 30-day mortality were 34.3% (43/105) and 1% (1/105), respectively. The lengths of postoperative hospital stays and incidence rates of complications were similar between the two matched groups (**Table 2**). None of the patients experienced intraperitoneal bleeding within 72 hours after surgery. In the NAR group, one patient developed bile leakage after hepatectomy and underwent percutaneous catheter drainage for two months. One patient in each group developed postoperative hepatic failure; after conservative treatment, the AR patient recovered, whereas the NAR patient died 25 days after surgery. Postoperative infection (definite positive after bacterial culture) occurred in three patients in each group; however, these patients recovered after treatment with antibiotics and immune regulation. Other common complications included pleural effusion and ascites, which occurred at similar rates between both groups and required ultrasound-guided percutaneous drainage. There were no significant differences between the two groups in the severity of complications according to the Clavien–Dindo classification.

**Table 2:** Comparison of postoperative outcomes.

Postoperative outcomes	NAR(n=57)	AR(n=48)	*P* value
	n (%)	n (%)	
30-day mortality	1(2.9)	0(0)	1.000
Postoperative hospital stay (days)	13(9-16)	12(8-17)	0.813
Overall complication	15(26.3)	11(22.9)	0.688
Infection	3(5.3)	3(6.3)	1.000
Bile leakage	1(1.8)	0(0)	1.000
Pleural effusion	5(8.7)	3(6.3)	0.724
Postoperative ascites	5(8.8)	4(8.3)	1.000
Liver failure	1(1.8)	1(2.1)	1.000
Severity of complication (Clavien–Dindo)			
Grade I-II	9(26.5)	8(23.5)	0.779
Grade III-IV	1(2.9)	1(2.9)	1.000

NAR, non-anatomical resection; AR, anatomical resection.

### Long-term survival

**Figure 2:**
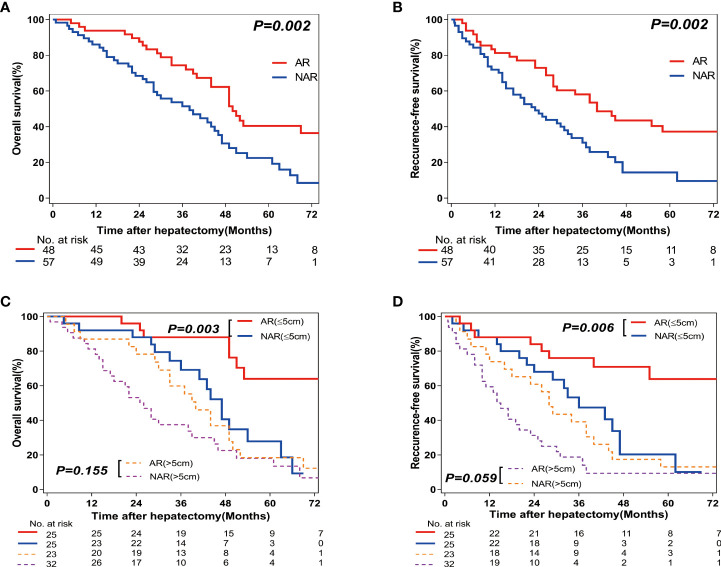
Overall survival (OS) and Recurrence-free survival (RFS) rates after Anatomic resection (AR) versus Non-anatomic resection (NAR) for combined hepatocellular-cholangiocarcinoma (cHCC-CCA) patients. **(A)** Overall survival (OS) and **(B)** recurrence-free survival (RFS) curves of cHCC-CCA patients in AR (n=57; *P*=0.002) and NAR (n=48; *P*=0.002) groups. **(C)** Overall survival (OS) and **(D)** recurrence-free survival (RFS) curves of cHCC-CCA patients with tumors ≤5 cm in size (n=50, *P*=0.006 and *P*=0.003, respectively) and >5 cm in size (n=55, *P*=0.059 and *P*=0.155, respectively).

A total of 105 patients were followed up for various periods (range=0.8–97 months; median=42 months). The 1-year, 3-year, and 5-year OS rates for all patients were 88.6%, 59.8%, and 29.0%, respectively. Correspondingly, the 1-year, 3-year, and 5-year RFS rates for all patients were 75.2%, 42.9%, and 22.8%, respectively. The 1-year, 3-year, and 5-year OS rates were significantly higher in the AR group as compared to those in the NAR group (91.7% vs 86.0%; 70.0% vs 51.1%; 36.8% vs 22.3%, respectively; *P*=0.002; **Figure 2A**). The 1-year, 3-year, and 5-year RFS rates were also higher in the AR group as compared to those in the NAR group (79.2% vs 71.9%; 56.0% vs 31.0%; 32.6% vs 14.4%, respectively; *P*=0.002; **Figure 2B**). **Tables 3**, **4** show the results of the stratified analyses (Cox proportional hazard regression analysis and log-rank test) for the predictors of RFS and OS rates. Univariate analyses revealed that the presence of HBsAg (positive vs negative) and cirrhosis (yes vs no), tumor nodularity (multiple vs solitary), tumor size (>5 cm vs ≤5 cm), resection type (AR vs NAR), surgical margin (R1 vs R0), differentiation (poor vs moderate/well), and the presence of lymph node metastasis and vascular invasion (yes vs no) were prognostic factors for RFS. Multivariate analyses revealed that the presence of: multiple tumors (hazard ratio [*HR*]=2.560, 95% confidence interval [*CI*]=1.346–4.868, *P*=0.004), larger tumors (>5 cm) (*HR*=2.036, 95% *CI*=1.174–3.534, *P*=0.011), AR (*HR*=0.573, 95% *CI*=0.334–0.982, *P*=0.043), lymph node metastasis (*HR*=3.043, 95% *CI*=1.348-6.869, *P*=0.007), and vascular invasion (*HR*=2.325, 95% *CI*=1.220–4.432, *P*=0.010) were significant predictors of RFS. Similarly, univariate analyses found that the presence of cirrhosis (yes vs no), tumor nodularity (multiple vs solitary), tumor size (>5 cm vs ≤5 cm), resection type (AR vs NAR), surgical margin (R1 vs R0), and the presence of lymph node metastasis and vascular invasion (yes vs no) were prognostic factors for OS. Multivariate analysis revealed that cirrhosis (*HR*=1.921, 95% *CI*=1.101–3.352, *P*=0.022), the presence of larger tumors (>5 cm) (*HR*=1.793, 95% *CI*=1.015–3.165, *P*=0.044), AR (*HR*=0.548, 95% *CI*=0.316–0.950, *P*=0.032), the presence of lymph node metastasis (*HR*=3.108, 95% *CI*=1.429–6.761, *P*=0.004), and vascular invasion (*HR*=3.544, 95% *CI*=1.831–6.862, *P*=0.001) were significant predictors of OS.

**Table 3:** Univariate and multivariate analyses of prognostic factors of recurrence-free survival.

Variable	Univariate analysis		Multivariate analysis	
	HR (95%CI)	*P* value	HR (95%CI)	*P* value
Age (≥60 vs <60years)	1.636 (0.967–2.766)	0.066		
Sex (female vs male)	0.812 (0.416–1.586)	0.543		
ECOG-PS (1 vs 0)	1.225 (0.719–2.089)	0.455		
HBsAg (positive vs negative)	1.661 (1.021–2.700)	**0.041**	1.368 (0.780–2.399)	0.275
AFP (>20 vs ≤20ng/ml)	1.088 (0.650–1.821)	0.749		
CEA (>5 vs ≤5ng/ml)	1.312 (0.742–2.320)	0.350		
CA199 (>37 vs ≤37U/L)	1.131 (0.714–1.791)	0.600		
Splenomegaly (yes vs no)	2.138 (1.297–3.523)	**0.003**	1.222 (0.652–2.288)	0.531
Child Pugh (B vs A)	0.514 (0.162–1.636)	0.260		
Tumor nodularity (multiple vs solitary)	5.132 (2.910–9.050)	**<0.001**	2.560 (1.346–4.868)	**0.004**
Tumor size (>5 vs ≤5cm)	2.852 (1.748–4.652)	**<0.001**	2.036 (1.174–3.534)	**0.011**
Procedure (laparoscopic vs. open)	0.834 (0.426–1.633)	0.596		
Resection (AR vs NAR)	0.475 (0.294–0.769)	**0.002**	0.573 (0.334–0.982)	**0.043**
Operation time (>180 vs ≤180mins)	0.881 (0.553–1.405)	0.596		
Blood loss (>500 vs ≤500ml)	0.681 (0.326–1.419)	0.305		
Transfusion (yes vs no)	0.657 (0.264–1.635)	0.367		
Surgical margin (R1 VS R0)	2.955 (1.255–6.958)	**0.013**	1.561 (0.581–4.195)	0.377
Differentiation (poor vs moderate/well)	1.762 (1.022–3.038)	**0.042**	1.236 (0.671–2.277)	0.496
Lymph node metastasis (yes vs no)	2.882 (1.354–6.133)	**0.006**	3.043 (1.348–6.869)	**0.007**
Vascular invasion (yes vs no)	3.661 (2.104–6.370)	**<0.001**	2.325 (1.220–4.432)	**0.010**

HR, hazards ratio; CI, confidence interval; HBsAg, hepatitis B surface antigen; AFP, α-fetoprotein; CEA, carcinoembryonic antigen; CA19-9, carbohydrate antigen 19-9; AR, anatomical resection; NAR, non-anatomical resection. Bold values: statistically significant P values.

**Table 4:** Univariate and multivariate analyses of prognostic factors of overall survival.

Variable	Univariate analysis		Multivariate analysis	
	HR (95%CI)	*P* value	HR (95%CI)	*P* value
Age (≥60 vs <60years)	1.301 (0.741–2.281)	0.359		
Sex (female vs male)	0.735 (0.364–1.487)	0.392		
ECOG-PS (1 vs 0)	1.082 (0.625–1.875)	0.778		
HBsAg (positive vs negative)	1.389 (0.849–2.274)	0.191		
AFP (>20 vs ≤20ng/ml)	1.327 (0.774–2.275)	0.304		
CEA (>5 vs ≤5ng/ml)	1.397 (0.776–2.514)	0.265		
CA199 (>37 vs ≤37U/L)	1.117 (0.696–1.793)	0.646		
Splenomegaly (yes vs no)	2.581 (1.552–4.294)	**<0.001**	1.921 (1.101–3.352)	**0.022**
Child Pugh (B vs A)	0.623 (0.196–1.984)	0.424		
Tumor nodularity (multiple vs solitary)	3.079 (1.886–5.164)	**<0.001**	1.515 (0.814–2.817)	0.190
Tumor size (>5 vs ≤5cm)	2.728 (1.650–4.510)	**<0.001**	1.793 (1.015–3.165)	**0.044**
Procedure (laparoscopic vs. open)	1.157 (0.586–2.283)	0.675		
Resection (AR vs NAR)	0.465 (0.284–0.761)	**0.002**	0.548 (0.316–0.950)	**0.032**
Operation time (>180 vs ≤180mins)	1.024 (0.635–1.653)	0.922		
Blood loss (>500 vs ≤500ml)	0.806 (0.385–1.688)	0.567		
Transfusion (yes vs no)	0.620 (0.249–1.544)	0.305		
Surgical margin (R1 VS R0)	3.736 (1.551–8.997)	**0.003**	2.024 (0.762–5.376)	0.157
Differentiation (poor vs moderate/well)	1.556 (0.895–2.703)	0.117		
Lymph node metastasis (yes vs no)	2.424 (1.149–5.112)	**0.020**	3.108 (1.429–6.761)	**0.004**
Vascular invasion (yes vs no)	4.103 (2.307–7.297)	**<0.001**	3.544 (1.831–6.862)	**<0.001**

HR, hazards ratio; CI, confidence interval; HBsAg, hepatitis B surface antigen; AFP, α-fetoprotein; CEA, carcinoembryonic antigen; CA19-9, carbohydrate antigen 19-9; AR, anatomical resection; NAR, non-anatomical resection. Bold values: statistically significant P values.

Since tumor size may be associated with prognosis, the patients were further classified into subsets according to tumor size: tumor size <5 cm (n=50) and >5 cm (n=55). In the patients with smaller tumors (<5 cm), higher RFS and OS rates were observed in the AR (n=25) group as compared with those in the NAR (n=25) group (*P*=0.006 and *P*=0.003, respectively; **Figures 2C, D**). In the patients with larger tumors (>5 cm), there were no differences in RFS and OS rates between the AR (n=23) and NAR (n=32) groups (*P*=0.059 and *P*=0.155, respectively; **Figures 2C, D**).

### Patient characteristics and long-term outcomes after PSM

After the 1:1 PSM, 68 patients were identified and classified into propensity-matched anatomical resection (PSM-AR) (n=34) and propensity-matched non-anatomical resection (PSM-NAR) groups (n=34) (**Table 1**). Except for the high laparoscopic resection rate in the PSM-AR group (26.5% vs 5.9%; *P*=0.045), there were no significant differences in demographic and clinicopathologic characteristics between the two groups after matching (**Table 1**). The operation time tended to be shorter in PSM-NAR (median time=180 in the PASM-NAR group as compared to 195 minutes for the PSM-NAR group; *P*=0.058).

**Figure 3:**
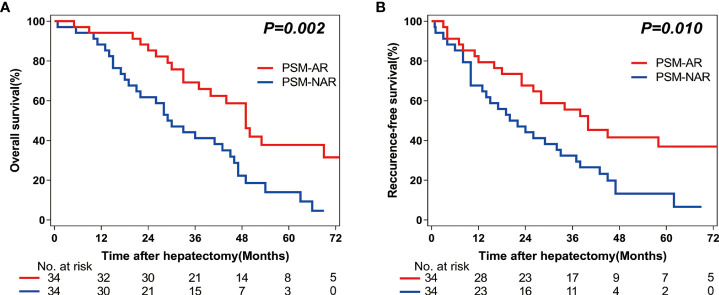
Overall survival (OS) and Recurrence-free survival (RFS) rates after Anatomic (AR) versus Non-anatomic resection (NAR) for combined hepatocellular-cholangiocarcinoma (cHCC-CCA) patients after propensity score matching (PSM). **(A)** Overall survival (OS) and **(B)** recurrence-free survival (RFS) curves of cHCC-CCA patients in PSM-AR (n = 34) and PSM-NAR (n = 34) groups after propensity score matching (PSM) (*P* = 0.002, P = 0.010, respectively).

Among the 68 patients included in this analysis, the 1-year, 3-year, and 5-year OS rates were higher in the PSM-AR group as compared to those in the PSM-NAR group (94.1% vs 88.2%; 65.9% vs 41.2%; 31.7% vs 14.0%, respectively; *P*=0.002; **Figure 3A**). The 1-year, 3-year, and 5-year RFS rates were also higher in the PSM-AR group as compared to those in the PSM-NAR group (79.4% vs 67.6%; 49.6% vs 32.4%; 30.2% vs 13.2%, respectively; *P*=0.010; **Figure 3B**).

## Discussion

The clinical significance of choosing AR or NAR in treating cHCC-CCA remains unclear because of the relative rarity of this primary liver malignancy, which has an incidence of 1.8% (118/6552; as observed in our study, which is consistent with previous reports) (6). In this single-center study, we have demonstrated that cHCC-CCA patients who underwent AR surgeries had longer DFS and OS times than those who underwent NAR surgeries, (both, before and after PSM analysis), especially for tumors <5 cm in diameter. To the best of our knowledge, this is the first report that compares the surgical outcomes of AR versus NAR in the treatment of cHCC-CCA; we find that patients who underwent AR, had better surgical outcomes than those who underwent NAR.

Although cHCC-CCA has features of both HCC and CC, several studies have observed that cHCC-CCA shares more etiological features with HCC than with iCCA, especially with respect to its epithelial characteristics (5, 15). In our study, the clinicopathological features of cHCC-CCA were more similar to those of patients suffering from HCC infected with HBV (hepatitis B virus), both of which are associated with elevated AFP levels in most patients. The results of this study are consistent with those of previous studies (15, 21). In clinical settings, cHCC-CCA is often misdiagnosed as either HCC or iCCA *via* imaging or hematology tests due to non-specific clinical manifestations, and a confirmed diagnosis of cHCC-CCA usually requires surgical resection (22). Since preoperative biopsy is not routinely used to diagnose cHCC-CCA (as large sampling areas are required and have low sensitivity of detection), some studies have explored the use of other risk factors to differentiate between cHCC-CCA and HCC or iCCA. Some of these factors include sex (men are more likely to develop cHCC-CCA than women), and the presence of chronic liver damage, cirrhosis, hepatitis infection, familial history of liver cancer, alcoholism, and diabetes (21, 23). Although both CA199 and AFP levels are expected to be higher than normal in cHCC-CCA patients, in this study, we found that elevated AFP levels were more common than elevated CA199 levels. Furthermore, 85.7% of cHCC-CCA patients in our study were men and 62.9% of them had HBV infections, which is consistent with previous reports (23, 24). Our results indicate that the clinicopathological characteristics of cHCC-CCA in the patients included in our study resemble those of HCC more than iCCA.

Surgical resection is widely accepted as an optimal curative treatment for cHCC-CCA and can provide patients with a chance of long-term survival (13, 25).The main objectives of surgical resectioning in treating cHCC-CCA are to completely remove the tumor, preserve sufficient residual liver volume for survival, and ensure negative resection margins. Unfortunately, until now, the prognostic differences in treating cHCC-CCA with either AR or NAR surgeries have not been reported. Usually, treatment with AR reduces tumor recurrence as it involves the removal of tumor-bearing portal vein branches and corresponding liver parenchyma. Since this supports long-term survival, several studies have reported that AR is superior to NAR for the treatment of HCC or iCCA with resection (17, 26, 27). NAR is considered to be beneficial for patients with cirrhosis or poorly preserved liver function (28). Since cHCC-CCA resembles both, HCC and iCCA, the long-term outcomes of surgical resection may be similar to those of HCC and iCCA; however, it is important to have data-backed proof of differences in surgical outcomes of cHCC-CCA patients after AR or NAR surgeries. Although there were no significant differences in the occurrences or types of postoperative complications between the two surgical methods, we found that AR is prognostically superior to NAR for cHCC-CCA treatment. Our results (both, before and after PSM) show that AR significantly improved the RFS and OS times for cHCC-CCA patients.

Multivariate analyses also showed that tumor size and nodularity, as well as the presence of lymph node metastasis and vascular invasion were independent risk factors for postoperative survival of cHCC-CCA patients; these patterns are consistent with those for patients with HCC or iCCA (29–33). Tumor size may influence surgical outcomes for HCC patients (34); this was shown in a large-scale study from Japan, which found that the recurrence rates for HCC patients with tumors of diameter 2–5 cm were significantly lower for those who underwent AR surgery rather than for those who underwent NAR surgery. However, there were no significant differences in surgical outcomes after liver resection for HCC tumors ≤2 cm or ≥5 cm in size between the AR and NAR groups (35). Due to the similarities between HCC and cHCC-CCA, tumor size can be expected to be a crucial risk factor for surgical outcomes of AR or NAR surgeries in cHCC-CCA patients. In this study, stratified analysis showed that AR provides a better long-term survival benefit than NAR for patients with tumors ≤5 cm in size. However, there were no significant differences in RFS and OS at 1, 3, and 5 years after resection surgery for patients with tumors >5 cm in size. One reason for this inconsistency could be that the cHCC-CCA tumors in the patients included in this study were more similar to CCA tumors than to HCC tumors; for CCA tumors, AR surgery provides no extra survival benefits over NAR surgery. Furthermore, the diameters of all the tumor masses in this study were >2.2 cm. Our results, therefore, suggest that AR should be recommended for cHCC-CCA patients with small tumors.

Despite our clear-cut results, this study has several limitations. Of these, one is that this study has a small sample size with all samples drawn from a single center. Second, this is a retrospective study, which means that there is a high chance of it having selection biases despite our use of PSM analysis. Third, we have not analyzed the impact of postoperative therapy on the long-term outcomes in patients due to unavailable data. In addition, not all patients included in this study underwent lymph node dissection, as several had normal lymph nodes (as observed by preoperative imaging). We recommend that more prospective studies with larger sample sizes and RCT studies be performed to fully evaluate the relative merits of AR and NAR surgeries in treating cHCC-CCA.

## Conclusion

In conclusion, the clinicopathologic characteristics of cHCC-CCA usually resemble those of HCC more than those of iCCA. Irrespective of the application of PSM, we found that AR was associated with better surgical outcomes as compared to NAR for patients with cHCC-CCA, especially for tumors of size ≤5 cm in diameter.

## Data availability statement

The original contributions presented in the study are included in the article/Supplementary Material. Further inquiries can be directed to the corresponding authors.

## Author contributions

Conception and design, W-QW, E-LZ, and S-HY. Analysis and interpretation of data, all authors. Drafting the article or revising it critically for important intellectual content, all authors. Final approval of manuscript, all authors. All authors contributed to the article and approved the submitted version.

## Funding

This work was partially supported by the National Natural Science Foundation of China (No.81902839).

## Acknowledgments

We thank Bullet Edits Limited for the linguistic editing and proofreading of the manuscript.

## Conflict of interest

The authors declare that the research was conducted in the absence of any commercial or financial relationships that could be construed as a potential conflict of interest.

## Publisher’s note

All claims expressed in this article are solely those of the authors and do not necessarily represent those of their affiliated organizations, or those of the publisher, the editors and the reviewers. Any product that may be evaluated in this article, or claim that may be made by its manufacturer, is not guaranteed or endorsed by the publisher.
